# Effective Reduction of SARS-CoV-2 RNA Levels Using a Tailor-Made Oligonucleotide-Based RNA Inhibitor

**DOI:** 10.3390/v14040685

**Published:** 2022-03-25

**Authors:** Veronika Nemethova, Petra Mazancova, Michal Selc, Kristina Jakic, Lucia Uhelska, Boglarka Nemethova, Alexandra Poturnayova, Lubos Drgona, Andrea Babelova, Filip Razga

**Affiliations:** 1 Selecta Biotech SE, Istrijska 6094/20, 841 07 Bratislava, Slovakia; mazancova@selectabiotech.com (P.M.); uhelska@selectabiotech.com (L.U.); b.nemethova@selectabiotech.com (B.N.); 2 Biomedical Research Center, Department of Nanobiology, Cancer Research Institute, Slovak Academy of Sciences, Dubravska Cesta 9, 845 05 Bratislava, Slovakia; michal.selc@savba.sk (M.S.); exonkiko@savba.sk (K.J.); andrea.babelova@savba.sk (A.B.); 3 Centre of Biosciences, Institute of Molecular Physiology and Genetics, Slovak Academy of Sciences, Dubravska Cesta 9, 840 05 Bratislava, Slovakia; alexandra.poturnayova@savba.sk; 4 Department of Oncohematology, Comenius University and National Cancer Institute, Klenova 1, 833 10 Bratislava, Slovakia; lubos.drgona@nou.sk

**Keywords:** SARS-CoV-2, COVID-19, RNA levels, oligonucleotide-based RNA inhibitor, RdRp

## Abstract

In only two years, the coronavirus disease 2019 (COVID-19) pandemic has had a devastating effect on public health all over the world and caused irreparable economic damage across all countries. Due to the limited therapeutic management of COVID-19 and the lack of tailor-made antiviral agents, finding new methods to combat this viral illness is now a priority. Herein, we report on a specific oligonucleotide-based RNA inhibitor targeting severe acute respiratory syndrome coronavirus 2 (SARS-CoV-2). It displayed remarkable spontaneous cellular uptake, >94% efficiency in reducing RNA-dependent RNA polymerase (RdRp) RNA levels in transfected lung cell lines, and >98% efficiency in reducing SARS-CoV-2 RNA levels in samples from patients hospitalized with COVID-19 following a single application.

## 1. Introduction

Since the emergence of severe acute respiratory syndrome coronavirus 2 (SARS-CoV-2) in 2019, the number of globally confirmed cases according to World Health Organization statistics exceeded 400 million across 222 countries by February 2022, with confirmed deaths of more than 5.7 million patients [1]. The worldwide paralysis caused by the coronavirus pandemic has triggered a global fight against this threat on several fronts. Preventive measures, vaccination, prophylaxis, secondary medical use of existing drugs, and symptomatic treatments are being applied to combat the virus [2,3,4,5,6]. The absence of a specific antiviral agent for those with serious health complications has placed pressure on scientists and research institutions around the world to develop effective agents against SARS-CoV-2.

SARS-CoV-2 is an enveloped positive-sense unsegmented single-stranded RNA virus, meaning that the base sequence of the RNA is recognized and treated as a later messenger RNA in the host’s cell. With a length of about 30 kb, the genome of SARS-CoV-2 is the largest RNA genome of all known RNA viruses. The replication of SARS-CoV-2 is operated by a set of non-structural proteins (nsps) that assemble into a multi-subunit polymerase complex. Among them, nsp12 is the catalytic subunit with RNA-dependent RNA polymerase (RdRp) activity [7,8]. RdRp plays a crucial role in the viral life cycle and is essential for viral survival; hence, targeting the corresponding RNA sequence to hinder its translation or induce its enzymatic degradation may be an effective therapeutic strategy. 

Therapeutic oligonucleotides have attracted great interest due to their potency and potential for changing the therapeutic landscape of many pathological conditions, including those of viral origin. Targeting conserved SARS-CoV-2 RNA sequences essential for viral replication offers a rational approach to inhibit viral infection and thereby halt disease progression [7,9]. The standard design of oligonucleotides is relatively straightforward as most modalities simply exploit knowledge of the primary sequence of the target RNA. However, the promiscuity of oligonucleotides, in terms of their binding to homologous sequences, places severe limitations on this approach since off-target-related toxicity undeniably contributes to the poor toxicological profile of these drugs [10]. In view of this persistent problem, the Oligonucleotide Safety Working Group unequivocally calls for increased safety and/or decreased toxicity of therapeutic oligonucleotides to achieve selective therapeutic action [11]. An unconventional platform first reported in 2017 also considered steric and thermodynamic aspects of the interactions between oligonucleotides and target RNA as additional parameters in the design of therapeutic oligonucleotides, which significantly improved their specificity and, hence, safety [12].

This proprietary platform, now called ESiNAR-X, served as basis for the development of a highly specific oligonucleotide-based lead pharmacological modality against SARS-CoV-2, namely ASC1R. It was designed to target the RNA region encoding the nsp12 catalytic subunit with RdRp activity. The present study demonstrates the antiviral potential of ASC1R in terms of its ability to reduce levels of RdRp RNA in transfected lung cell lines by more than 94% and levels of SARS-CoV-2 RNA in patient samples by more than 98% within 24 h after a single application.

## 2. Materials and Methods

### 2.1. Oligonucleotides

ASC1R, a 37mer phosphorothioate DNA with an internal poly(ethylene glycol) (PEG) linker, was synthesized by Selecta Biotech (Bratislava, Slovakia) using an automated H-28 DNA synthesizer (K&A Laborgeraete, Schaafheim, Germany). The oligonucleotide was labeled with Cyanine 5 (Cy5) and biotin at the 5’ and 3’ ends, respectively, to generate 5’-Cy5-CAAATGTTAAAAACACTAT(PEG_12_)GCAGTTGTGGCATCTCCT-biotin. The high-performance liquid chromatography-purified compound was stored at −20 °C in either lyophilized form or dissolved in nuclease-free water.

A 119 nt single-stranded DNA (5’-CCCA^AGCTTGTGAAATGGTCATGTGTGGCGGTTCACTATATGTTAAACCAGGTGGAACCTCATCAGGAGATGCCACAACTGCTTATGCTAATAGTGTTTTTAACATTTGG^AATTCCG) corresponding to the target RdRp region, including overhang sequences from a 5’ *Hind*III and a 3’ *Eco*RI restriction site, was synthesized by GeneLink (Orlando, FL, USA) for cloning purposes.

Primers for the amplification of the 119 nt DNA sequence for cloning purposes (Fw, 5’-CCCAAGCTTGTGAAATGGTCATGTGTG; Rev, 5’-CGGAATTCCAAATGTTAAAAACACTA) and primers and probe for the quantification of the RdRp region (Fw, 5’-GTGAAATGGTCATGTGTGGCGG; Rev, 5’-CAAATGTTAAAAACACTATTAGCATA; Probe, 5’-FAM-CAGGTGGAACCTCATCAGGAGATGC-BBQ) were purchased from Metabion International AG (Planegg, Germany).

A CE IVD gb SARS-CoV-2 Multiplex Kit for RT-qPCR analysis of the E gene was purchased from Generi Biotech (Hradec Králové, Czech Republic).

An Hs01060665_g1 Assay Kit for RT-qPCR analysis of the b-actin housekeeping gene was purchased from Thermo Fisher Scientific (Waltham, MA, USA).

### 2.2. Hybridization

ASC1R and the 119 nt target RdRp sequence were mixed in an equimolar ratio (10^12^ molecules each) and incubated in a hybridization buffer comprising 0.001% sodium dodecyl sulfate (SDS; Sigma Aldrich, Darmstadt, Germany), 0.1 M NaCl (Centralchem, Bratislava, Slovakia), and 1 mM EDTA (Centralchem) for 30 min at 37 °C. The resultant heteroduplexes, control ASC1R (10^12^ molecules), and RdRp sequence (10^13^ molecules) were then loaded onto a 6% polyacrylamide gel and subjected to electrophoresis at 110 V (40 min, room temperature). Afterwards, nucleic acids were stained with GelRed Nucleic Acid Stain (Millipore, Darmstadt, Germany) for 20 min and visualized at 590 nm, and Cy5-labeled ASC1R was visualized at 680 nm, both using a ChemiDoc MP instrument (Bio-Rad Laboratories, Hercules, CA, USA).

### 2.3. Vector Construction

The 119 nt target RdRp sequence was amplified using high-fidelity DNA polymerase and Phusion Plus PCR Master Mix (Thermo Fisher Scientific, Waltham, MA, USA) to ensure high accuracy during DNA synthesis, and amplicons were purified using a QIAquick PCR Purification Kit (Qiagen, Germantown, MD, USA). Purified amplicons (1 µg) and 5 µg of pcDNA 3.1 vector (Invitrogen, Waltham, MA, USA) were cleaved using FastDigest *Hind*III and *Eco*RI (Thermo Fisher Scientific) according to the manufacturer’s instructions. Products were separated on a 1.5% agarose gel (110 V, 40 min, room temperature), excised, and purified using a QIAEX II Gel Extraction Kit (Qiagen). Ligation was performed overnight at 4 °C using T4 DNA ligase (Roche, Grenzach-Wyhlen, Germany) at a 1:3 molar ratio of vector to insert.

### 2.4. Bacterial Transformation and Vector Cloning

NEB 5-alpha competent *Escherichia coli* cells (New England BioLabs, Hitchin, UK) were transformed with the vector according to the manufacturer’s instructions. Briefly, 10 ng of plasmid DNA was carefully mixed with cells and incubated on ice for 30 min. Cells were heat shocked at 42 °C for 30 s and chilled on ice for 5 min. After the addition of 950 µL of SOC medium (Sigma Aldrich, St. Louis, MO, USA), the mixture was shaken at 200 rpm for 60 min at 37 °C, and the cells were diluted and plated on selective LB agar (Invitrogen, Waltham, MA, USA) plates containing 100 µg/mL ampicillin (Gibco, Waltham, MA, USA). After incubation overnight at 37 °C, selected colonies were inoculated into 3 mL of fresh LB medium containing 100 µg/mL ampicillin and incubated overnight at 37 °C with shaking at 200 rpm. The vector construct with the RdRp sequence was verified by Sanger sequencing. Plasmids for subsequent transfection of lung cells were purified using a PureLink HiPure Plasmid Miniprep Kit (Invitrogen, Waltham, MA, USA).

### 2.5. Cell Culture

A primary cell line of human embryo lung cells (HEL299) and stable adenocarcinomic human alveolar basal epithelial cells (A549) were used in this study. HEL299 and A549 cells were maintained in Minimum Essential Medium (MEM; Gibco, Waltham, MA, USA) and low-glucose Dulbecco’s Modified Eagle’s Medium (DMEM; Gibco) supplemented with 10% fetal bovine serum (FBS; Gibco, Waltham, MA, USA) and 1% penicillin/streptomycin (P/S; Gibco, Waltham, MA, USA) at 37 °C with 5% CO_2_. Before experiments, the amount of FBS was gradually decreased to 2% to slow cell growth and avoid contact inhibition.

### 2.6. Transfection of Cells

HEL299 and A549 cells were seeded 1 day before transfection in 48-well plates in 200 µL MEM and low-glucose DMEM supplemented with 2% FBS and 1% P/S at a density of 1 × 10^5^ cells/well. Cells were transfected using ViaFect (Promega, Madison, WI, USA) according to the manufacturer’s instructions. Specifically, the transfection reagent and plasmid were pre-mixed at a ratio of 1.5:1 *v*/*w* and incubated for 15 min at room temperature. The transfection complex was then added to cells and incubated for 24 h at 37 °C with 5% CO_2_. HEL299 cells, as a primary cell line, were used for experiments immediately after transfection due to their limited proliferative capacity [13]. Transfected A549 cells were selected and maintained using Geneticin (Gibco, Waltham, MA, USA) at a concentration of 600 µg/mL for 14 days. Geneticin-resistant cells were used in subsequent experiments.

### 2.7. Treatment of Cells

For confocal microscopy, transfected HEL299 and A549 cells were seeded onto glass slides at a density of 2 × 10^4^ cells/slide, exposed to 0.005–10 µM of Cy5-labeled ASC1R, and incubated at 37 °C and 5% CO_2_ for 24 h. For the quantification of cellular uptake, transfected HEL299 and A549 cells (seeded in 48-well plates in 200 µL of media at a density of 5 × 10^4^ cells/well) were exposed to 0.005–10 µM of Cy5-labeled ASC1R and incubated at 37 °C and 5% CO_2_ for 24 h. To analyze the efficacy of ASC1R, transfected HEL299 and A549 cells (seeded in 48-well plates in 200 µL of media at a density of 5 × 10^4^ cells/well) were exposed to 5 µM of Cy5-labeled ASC1R and incubated at 37 °C and 5% CO_2_ for 24 h. Non-treated cells served as controls and were incubated in parallel.

### 2.8. Confocal Microscopy

After treatment, cells on glass slides were washed with phosphate-buffered saline (PBS; Oxoid, Lenexa, KS, USA) to remove extracellular ASC1R, and a fresh medium without Phenol Red was added. Cell membranes were stained with FM 4-64FX membrane stain (Invitrogen, Waltham, MA, USA) and visualized at 744 nm. Intracellular ASC1R was visualized at 680 nm using a Leica TCS SP8 AOBS confocal microscope (Leica Microsystems, Wetzlar, Germany).

### 2.9. Quantification of Cellular Uptake

After treatment, cells were trypsinized, pelleted at 200 × *g*, and washed with PBS to remove extracellular ASC1R. Cells (5 × 10^4^) were lysed using 40 µL of lysis buffer containing RIPA (Serva, Heidelberg, Germany) and 10% SDS at 4 °C for 30 min, and then briefly sonicated using a Sonopuls Mini 20 instrument (Bandelin, Germany). A 10 µL sample of cell lysate was loaded onto a 6% polyacrylamide gel and subjected to electrophoresis (110 V, 40 min, room temperature). ASC1R was visualized at 680 nm, and the amount of ASC1R was normalized against a standard and expressed as the number of ASC1R molecules per single cell.

### 2.10. Collection of Patient Samples

Twenty-six biological specimens were obtained from patients diagnosed with COVID-19 using nasopharyngeal swabs collected in 1.5 mL microcentrifuge tubes containing 1 mL of MEM supplemented with 2% FBS. The study was approved by the Ethics Committee of the National Cancer Institute in Bratislava, Slovakia, and written informed consent was provided by all patients. All procedures were performed in compliance with relevant laws and institutional guidelines and in accordance with the ethical standards of the Declaration of Helsinki.

### 2.11. Treatment of Patient Samples

Swabs were squeezed into microcentrifuge tubes, vigorously vortexed, removed from the tubes, and properly disposed. Cells were pelleted at 300 × *g*, resuspended in 400 µL of MEM supplemented with 2% FBS, and divided into two equal aliquots (200 µL each). One aliquot was exposed to 5 µM of Cy5-labeled ASC1R and incubated at 37 °C and 5% CO_2_ for 24 h, while the other served as a non-treated control and was incubated under the same conditions in parallel.

### 2.12. RNA Isolation

After treatment, HEL299 and A549 cells were trypsinized, pelleted at 200× *g*, and washed with PBS to remove extracellular ASC1R. Treated patient samples were pelleted at 300× *g* and washed with PBS. RNA was isolated using *RNA*GEM (MicroGEM, Southampton, UK) following the manufacturer’s instructions. Non-treated parallels were processed identically.

### 2.13. Real-Time RT-PCR

Real-time RT-PCR was carried out in a final volume of 20 µL containing 2 µL of RNA using AmpliTune 1-step RT-qPCR Probe Mix (Selecta Biotech, Bratislava, Slovakia)according to the instructions for users. Amplification was carried out on an AriaMX automated PCR instrument (Agilent, Santa Clara, CA, USA). Briefly, RNA was reverse-transcribed at 50 °C for 10 min, whereas subsequent cDNA amplification involved the initial activation of DNA polymerase at 95 °C for 3 min and 40 cycles of denaturation (95 °C for 15 s) and annealing/elongation (58 °C for 30 s). All data were normalized against b-actin. Data from treated cells were also normalized against controls and analyzed by the 2^-ΔΔCt^ method.

## 3. Results and Discussion

### 3.1. Binding of ASC1R to the Target SARS-CoV-2 RNA Region

**Figure 1:**
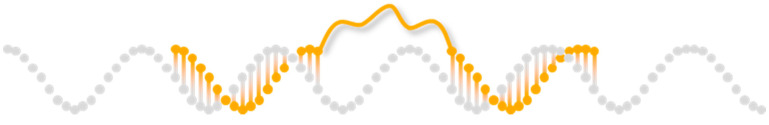
Schematic representation of the annealing of ASC1R (orange) to the target SARS-CoV-2 RNA (gray).

The fundamental requirement for the therapeutic action of any oligonucleotide-based modality is its ability to bind to the target RNA. ASC1R has an unconventional design with an internal linker between the recognition sequences of the inhibitor (Figure 1); hence, we considered it necessary to demonstrate that the mere presence of the linker does not cause obstructions in complementarity-driven annealing of ASC1R to the target RNA.

**Figure 2:**
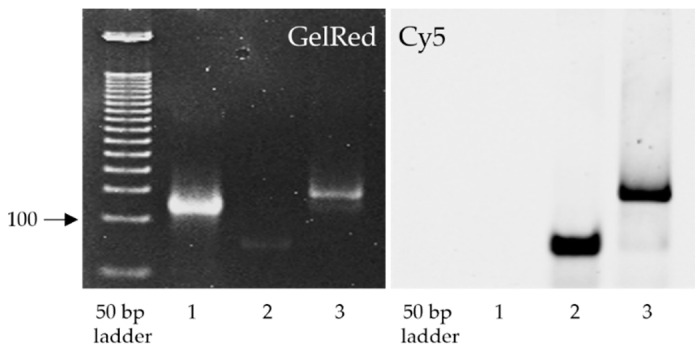
Binding of ASC1R to the target RdRp sequence. Lane 1, Control 119 nt RdRp sequence; Lane 2, Control ASC1R; Lane 3, ASC1R-RdRp heteroduplex. The polyacrylamide gel was imaged using filters for GelRed (**left**) and Cy5 (**right**).

As shown in Figure 2, ASC1R binds to the 119 nt target sequence corresponding to the RdRp region of SARS-CoV-2, forming a stable heteroduplex. This is evidenced by a moderate decrease in the electrophoretic mobility of the RdRp sequence detected after nucleic acid staining (Figure 2, left, Lanes 1 versus 3). Furthermore, the same band in Lane 3 was visible under a Cy5 filter used for the detection of Cy5-labeled ASC1R (Figure 2, right).

### 3.2. Cellular Internalization of ASC1R

**Figure 3:**
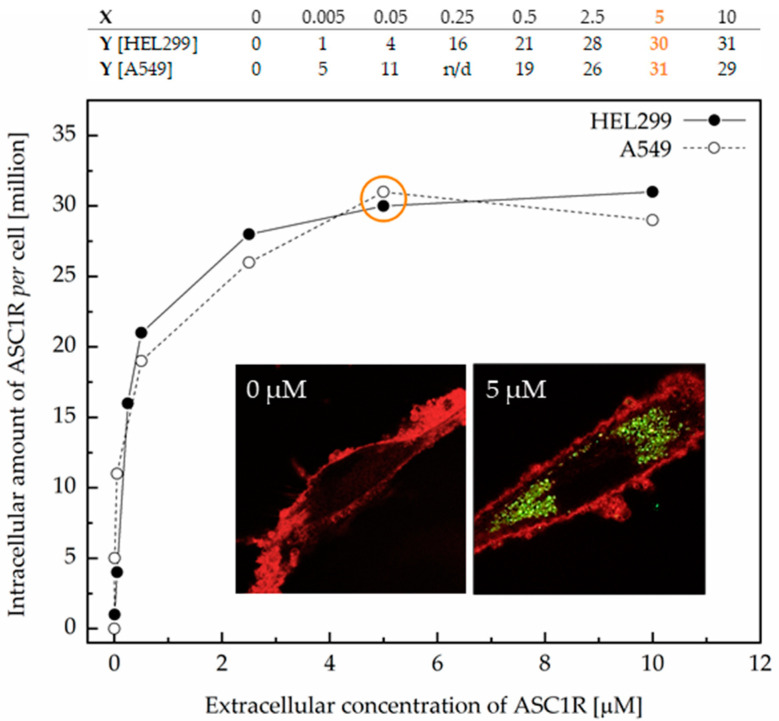
Internalization profile of ASC1R in transfected HEL299 and A549 cells. The insets show representative confocal microscopy images of HEL299 cells with the cell membrane colored red and ASC1R colored green.

One of the major problems hindering the smooth translation of therapeutic oligonucleotides into clinical practice is their unsatisfactory cellular uptake [9]. For this reason, ASC1R was subjected to internalization experiments before functional testing, and the results were evaluated by confocal laser scanning microscopy and electrophoresis. Transfected HEL299 and A549 cells were exposed to an increasing concentration of ASC1R ranging between 0.005 and 10 µM to investigate the internalization profile of the inhibitor. The results showed that the intracellular concentration of ASC1R reached a plateau of 30 million molecules per cell at an applied concentration of 5 µM (Figure 3), an increase of up to 100-fold over other state-of-the-art oligonucleotides [14,15,16,17]. The amount of internalized inhibitor was comparable in both cell lines. ASC1R was detected solely in the cytoplasm and was absent in the nucleus. Importantly, cellular internalization was completely spontaneous (i.e., without the use of any transfection reagent or supporting method, such as electroporation). Of note, none of the applied concentrations of ASC1R affected cell viability, which varied between 94% and 100% (data not shown). With respect to the observed plateau in cellular uptake, 5 µM ASC1R was selected for its subsequent functional validation in vitro and ex vivo.

### 3.3. Efficacy of ASC1R in Transfected HEL299 and A549 Cells

**Figure 4:**
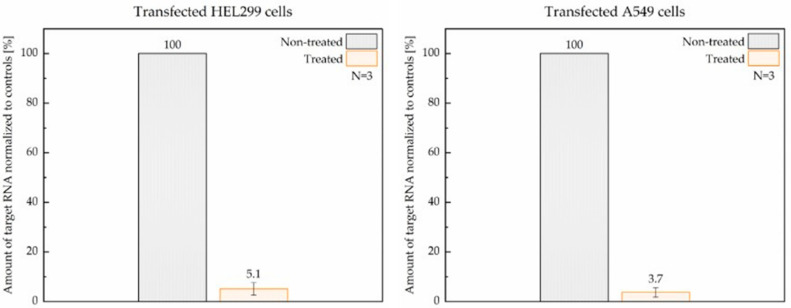
Reduction in RdRp RNA levels upon treatment with ASC1R in transfected HEL299 (**left**) and A549 (**right**) cells assessed by RT-qPCR. Non-treated parallels served as controls. Transfection and treatment of cells were repeated three times.

Transfected lung cells treated with 5 µM ASC1R and non-treated controls were incubated in parallel for 24 h. The efficacy of ASC1R was evaluated as the percentage difference between the amounts of expressed RdRp RNA in treated and non-treated cells post-incubation. Raw Ct values for non-treated HEL299 and A549 parallels were 18.7, 19.6, and 20.0 and 20.5, 24.5, and 27.8, respectively. As shown in Figure 4, after a single application of 5 µM ASC1R, the amount of RdRp RNA measured by RT-qPCR decreased by >94% on average.

### 3.4. Efficacy of ASC1R in Patient Samples

**Figure 5:**
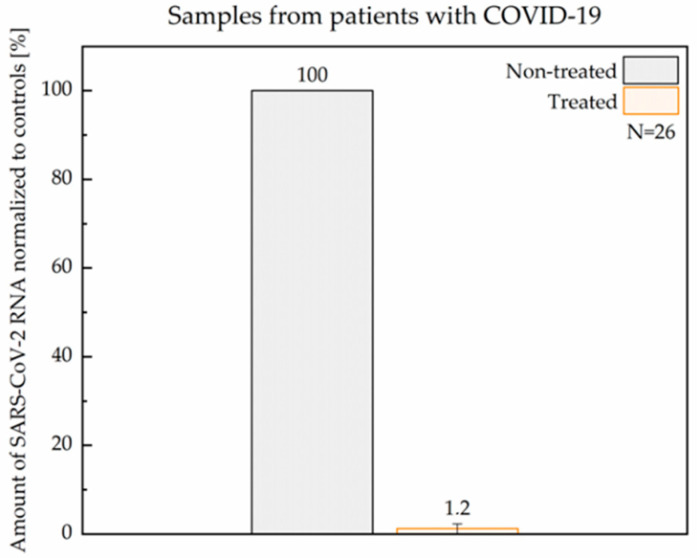
Reduction in SARS-CoV-2 RNA level upon treatment with ASC1R in samples from patients with COVID-19 assessed by RT-qPCR. Non-treated parallels served as controls.

Twenty-six nasopharyngeal samples from patients diagnosed with COVID-19 were used in this study. Each sample was handled, treated, and evaluated independently. Equal aliquots of non-treated and ASC1R-treated cells derived from each patient sample were incubated in parallel for 24 h. Similarly to transfected cells, the efficacy of ASC1R was evaluated as the percentage difference in the amount of SARS-CoV-2 RNA post-incubation. The median raw Ct values for non-treated parallels after a 24 h incubation was 24.7 (*N* = 26; Q25–Q75 range = 22.0–28.8). As shown in Figure 5, after a single application of 5 µM ASC1R, SARS-CoV-2 RNA levels measured by RT-qPCR decreased by >98% on average (Table S1). The loss of viral RNA was confirmed by the simultaneous analysis of the E gene as an independent locus of the non-segmented SARS-CoV-2 genome. A proportional reduction in the E gene in ASC1R-treated samples (data not shown) indicates that the binding of ASC1R to the viral RNA triggered its degradation, presumably due to the RNase H-mediated enzymatic cleavage of the formed DNA-RNA duplex [18].

## 4. Conclusions

The persistent coronavirus pandemic and the high mortality associated with COVID-19 demand urgent solutions. In this study, we reported the antiviral potential of a tailor-made oligonucleotide-based inhibitor targeting SARS-CoV-2, which is capable of spontaneously penetrating the cells, binding to viral RNA, and inducing its enzymatic degradation with a remarkable > 98% efficacy after a single application. The therapeutic potential of ASC1R demonstrated herein could translate into substantial clinical benefits for patients with COVID-19. Furthermore, in the context of infectious diseases, our results provide implications for the research and development of analogous antivirals for other diseases of viral origin. The findings could help to meet the global challenge of developing new and safe treatment modalities.

## Data Availability

Not applicable.

## Supplementary Materials

The following supporting information can be downloaded at: https://www.mdpi.com/article/10.3390/v14040685/s1, Table S1: Ct values of RdRp and b-actin and relative quantification of RdRp in patient samples.
