# Supplementary material for: Effective Reduction of SARS-CoV-2 RNA Levels Using a Tailor-Made Oligonucleotide-Based RNA Inhibitor
Source: Viruses. 2022 Mar 25;14(4):685. doi: 10.3390/v14040685 (PMC9029688; doi:10.3390/v14040685)
Supplement: Supplementary file 1 [file viruses-14-00685-s001.zip › Table S1.pdf]

Table S1. Ct values of RdRp and β-actin and relative quantification of RdRp in patient samples.

ID	Non-treated		Treated		$2^{-\Delta\Delta Ct}$	
	Ct RdRp	Ct β-actin	Ct RdRp	Ct β-actin	fold	%
1	19.7	29.3	24.7	29.5	0.035	3.5
2	22.8	30.6	28.1	30.7	0.027	2.7
3	23.9	30.8	30.4	31.0	0.013	1.3
4	22.7	29.1	30.0	29.5	0.009	0.9
5	17.8	29.1	23.6	29.3	0.021	2.1
6	25.2	30.5	30.8	31.1	0.031	3.1
7	28.0	30.8	n/d	31.7	ND	0
8	33.6	31.1	n/d	32.2	ND	0
9	27.3	30.5	34.5	30.8	0.008	0.8
10	21.8	29.8	27.1	30.2	0.033	3.3
11	26.0	30.0	32.6	30.1	0.011	1.1
12	30.7	30.8	n/d	31.0	ND	0
13	20.7	29.1	27.6	30.4	0.020	2.0
14	23.2	31.2	n/d	31.8	ND	0
15	29.1	30.3	35	30.5	0.019	1.9
16	32.2	31.4	n/d	31.7	ND	0
17	19.5	29.5	25.3	29.7	0.021	2.1
18	25.4	29.8	32.7	30.4	0.010	1.0
19	27.2	30.7	n/d	31.1	ND	0
20	24.1	31.4	30.0	31.5	0.018	1.8
21	32.7	30.9	n/d	31.3	ND	0
22	21.1	29.7	28.4	29.9	0.007	0.7
23	18.6	29.6	25.1	30.1	0.015	1.5
24	24.2	31.3	30.8	31.4	0.011	1.1
25	30.6	30.2	n/d	31.6	ND	0
26	29.7	30.4	n/d	30.2	ND	0

n/d - not detected

ND - not determined
